# A Multicenter Study of 239 Patients Aged Over 70 Years With Diffuse Large B-Cell Lymphoma in China

**DOI:** 10.3389/fphar.2022.953808

**Published:** 2022-07-18

**Authors:** Chunli Yang, Qiaoer Li, Ke Xie, Yakun Zhang, Dania Xiang, Yunwei Han, Liqun Zou

**Affiliations:** ^1^ State Key Laboratory of Biotherapy and Cancer Center, West China Hospital, Sichuan University, Chengdu, China; ^2^ Department of Oncology, West China Hospital, Sichuan University, Chengdu, China; ^3^ Department of Oncology, Sichuan Academy of Medical Sciences and Sichuan Provincial People’s Hospital, Chengdu, China; ^4^ Department of Oncology, Affiliated Hospital of North Sichuan Medical College, Nanchong, China; ^5^ Issaquah High School, Issaquah, WA, United States; ^6^ Department of Oncology, Affiliated Hospital of Southwest Medical University, Luzhou, China

**Keywords:** diffuse large B-cell lymphoma, elderly, survival, prognosis, treatment

## Abstract

**Background:** Diffuse large B-cell lymphoma (DLBCL) is the most common aggressive lymphoma subtype worldwide and occurs frequently in the elderly population. However, there are limited data on the clinical profiles of patients with DLBCL over 70 years of age. Our objective was to summarize the clinical characteristics, treatment strategies and survival outcomes of this population in China.

**Methods:** This multicenter retrospective study was conducted in China from January 2012 to July 2020 to investigate the clinical characteristics and survival outcomes. A total of 239 patients with DLBCL aged over 70 years underwent pretreatment evaluations, treatment, and follow-up at local hospitals. The primary endpoints were the progression-free survival (PFS) and the overall survival (OS) rates at 2 years. Secondary endpoints included median PFS and OS, the estimated PFS and OS rates at 5 years, and adverse events during treatment.

**Results:** With a median follow-up of 50 months (range, 1–102 months), the 2-year PFS and OS rates were 53.0% and 65.5%, respectively. The median PFS and OS were 42.1 and 96.4 months, respectively; and the estimated 5-year PFS and OS rates were 44.7% and 56.1%, respectively. Hematological toxicities were the most common adverse effects in this study, accounting for 90.4%; and leukopenia was the most frequently observed ≥ grade 3 event. Furthermore, we found that regimens without rituximab and chemotherapy cycles < 6 were significantly associated with worse survival. Additionally, in the 70–80-year group, reduction in chemotherapy dose was associated with a significantly shorter OS, with a 2-year OS rate of 74.4% in the full dose group, compared to 67.1% for the decreased-dose group (*p* = 0.044).

**Conclusion:** Our study presents the clinical profiles and survival outcomes of elderly patients with DLBCL in China. Treatment of these patients requires careful evaluation of toxicities and benefits. To this end, a prognosis model, such as comprehensive geriatric assessment, is required in clinical practice to optimally manage elderly patients with DLBCL.

## Introduction

Diffuse large B-cell lymphoma (DLBCL) is the most common aggressive lymphoma subtype worldwide, accounting for 30–40% of non-Hodgkin lymphoma (Al- Hamadani et al., 2015). From the Surveillance, Epidemiology, and End Results Program (SEER) database, the incidence of DLBCL increases with age; the median diagnosis age is 66 years. Compared to patients with DLBCL aged younger than 60–70 years, older patients had a worse prognosis (Project TIN- HsLPF, 1993; Zhou et al., 2014; Choi et al., 2018). Moreover, most clinical trials have excluded these patients, which has led the need for elucidation of optimal treatment options and survival outcomes in the real world for patients over 70 years of age.

R-CHOP, known as combined regimen rituximab, cyclophosphamide, doxorubicin, vincristine, prednisone, is the standard regimen for initial treatment in DLBCL(Tilly et al., 2015; NCCN, 2022), reaching a cure rate of 60% for the entire cohort (Nowakowski et al., 2016). However, most elderly patients present in poor physical condition and are frequently accompanied by other diseases, which limit the use of a standard dose of the regimen and also present a worse impact on survival. Therefore, dose-reducing regimens or non-anthracycline-containing treatments have been explored in clinical trials, such as R-miniCHOP, R-GemOx (rituximab, gemcitabine, oxaliplatin), and R2 (rituximab, lenalidomide) (Peyrade et al., 2011; Shen et al., 2018; Gini et al., 2019), which achieved a balance of efficacy and tolerance in selected elderly patients with DLBCL. In China, the 5-year OS rate was reported to be 51.9% for DLBCL patients aged over 60 years between 2006 and 2012 in Beijing, including a sample of 349 patients, and 42.8% (145/349) for DLBCL patients aged over 70 years (Liu et al., 2019). To date, there have been a limited number of studies on the clinical profiles of patients with DLBCL over 70 years of age.

In this study, we enrolled 239 patients with DLBCL aged 70 years and over from five hospitals in China to summarize the clinical characteristics, analyze survival outcomes, and examine factors influencing prognosis.

## Materials and Methods

We retrospectively reviewed the records of all newly diagnosed DLBCL patients who were over 70 years old at five hospitals in China from January 2012 to July 2020, including West China Hospital, Sichuan University; Sichuan Cancer Hospital and Institute; Sichuan Provincial People’s Hospital; Affiliated Hospital of North Sichuan Medical College and the Affiliated Hospitals of Southwest Medical University. For all cases, the pathological diagnosis was confirmed by expert hematopathologists in these five hospitals according to the World Health Organization classification of hematopoietic and lymphoid tumors (Swerdlow et al., 2017). Critical exclusion criteria were age less than 70 years and involvement of the central nervous system lymphoma at diagnosis.

All patients underwent pre-treatment evaluations, treatment, and follow-up at the local hospital mentioned above. The evaluations included demographics of the patients, blood tests, serum lactate dehydrogenase (LDH), and detection of bone marrow biopsy. The staging was determined according to the Ann Arbor staging system, and efficacy of the treatment was evaluated using enhanced computed tomography (CT) or positron emission tomography (PET-CT). The Hans algorithm was applied to divide patients into germinal center B cell (GCB) and non-GCB subtypes. Bulky disease was defined as any mass diameter exceeding 7 cm. The investigators also reviewed therapy regimens, cycles, and adverse events. The reduced dose chemotherapy in this study was defined by a 20–50% decrease in the standard dose.

The primary endpoints of this study were the PFS and the OS rates at 2 years. Secondary endpoints included median PFS and OS, 5-year PFS and OS rates, and adverse events during treatment. The PFS was calculated from diagnosis to disease progression, recurrence, any cause of death, or last follow-up; and OS was from diagnosis to death for any reason or last follow-up.

We summarized PFS and OS using the Kaplan-Meier method and compared differences by the log-rank test. Univariate logistic regression analysis evaluated the variables in predicting survival for patients with DLBCL. Parameters identified as statistically significant risk factors were assessed in multivariate logistic regression analysis. Survival data was analyzed using SPSS version 25.0 (IBM Corp., Armonk, NY), and a two-sided *p* < 0.05 was considered a statistically significant. The study was conducted in accordance with regulatory requirements and was approved by the Ethics Committee of the West China Hospital of Sichuan University.

## Results

### Patient Characteristics

We identified 239 newly diagnosed patients with DLBCL aged over 70 years from January 2012 to July 2020. The clinical characteristics are listed in Table 1. The median age at diagnosis was 75.4 years (range, 70–91 years), and the ratio of males to females was 1.5:1. Stage III/IV patients accounted for 59.8% and 27.2% had extranodal involvement at more than one site. There were 200 patients who with a classified subtypes using the Hans algorithm, 21.8% for the GCB subtype, 61.9% for the non-GCB subtype, and 16.3% of the patients remained with unknown subtype. According to the IPI score (Project TIN- HsLPF, 1993), 25.5% of the cases were evaluated as belonging to the high-risk group. Approximately 85.8% of patients had other diseases.

**TABLE 1:** Clinical characteristics of 239 patients with DLBCL at diagnosis.

Parameters	All patients n = 239	70–79 years n = 200	≥80 years n = 39
Median age	75.4 (70-91)	74.0 (70-79)	82.6 (80-91)
**Sex**			
Male	143 (59.8%)	117 (58.5%)	26 (66.7%)
Female	96 (40.2%)	83 (41.5%)	13 (33.3%)
**ECOG score**			
0–1	193 (80.8%)	163 (81.5%)	30 (76.9%)
≥2	46 (19.2%)	37 (18.5%)	9 (23.1%)
**B symptoms**			
Yes	174 (72.8%)	144 (72.0%)	30 (76.9%)
No	65 (27.2%)	56 (28.0%)	9 (23.1%)
**Subtype**			
GCB	52 (21.8%)	44 (22.0%)	8 (20.5%)
non-GCB	148 (61.9%)	122 (61.0%)	26 (66.7%)
Unknown	39 (16.3%)	34 (17.0%)	5 (12.8%)
**Ki-67 index**			
>90%	7 (2.9%)	6 (3.0%)	1 (2.6%)
≤90%	192 (80.3%)	162 (81.0%)	30 (76.9%)
Unknown	40 (16.7%)	32 (16.0%)	8 (20.5%)
**Number of extra-nodal involvement**			
0 or 1	174 (72.8%)	150 (75.0%)	24 (61.5%)
≥2	65 (27.2%)	50 (25.0%)	15 (38.5%)
**Bone marrow Involvement**			
Yes	12 (5.0%)	11 (5.5%)	1 (2.6%)
No	227 (95.0%)	189 (94.5%)	38 (97.4%)
**Ann Arbor stage**			
I-II	96 (40.2%)	84 (42.0%)	12 (30.8%)
III-IV	143 (59.8%)	116 (58.0%)	27 (69.2%)
**Bulky disease**			
Yes	38 (15.9%)	33 (16.5%)	5 (12.8%)
No	201 (84.1%)	167 (83.5%)	34 (87.2%)
**Serum LDH**			
Normal	102 (42.7%)	85 (42.5%)	17 (43.6%)
Elevated	137 (57.3%)	115 (57.5%)	22 (56.4%)
**IPI score**			
1	52 (21.8%)	44 (22.0%)	8 (20.5%)
2	61 (25.5%)	55 (27.5%)	6 (15.4%)
3	65 (27.2%)	54 (27.0%)	11 (28.2%)
4-5	61 (25.5%)	47 (23.5%)	14 (35.9%)
**Comorbidities**			
Yes	205 (85.8%)	171 (85.5%)	34 (87.2%)
No	34 (14.2%)	29 (14.5%)	5 (12.8%)

DLBCL, diffuse large B cell lymphoma; ECOG, eastern cooperative oncology group; GCB, germinal center B-cell like; LDH, lactate dehydrogenase; IPI, international prognostic index.

### Treatment and Response

As presented in Table 2, 228 patients received initial treatment, including 91.6% receiveing chemotherapy, and 3.8% radiotherapy or surgery, while 4.6% of patients received no therapy. In total, there were 219 patients received chemotherapy alone or with rituximab (Table 3); 1,029 total treatment cycles were administered and the median number of cycles for each patient was 4.7 (range, 1-6 cycles). The most common chemotherapy regimen applied was CHOP (cyclophosphamide, doxorubicin, vincristine, prednisone), accounting for 84.5%, and other regimens including CVP (cyclophosphamide, vincristine, prednisone), BR (bendamustine, rituximab), oral etoposide or cyclophosphamide. Rituximab was not administered in 28.8% of the patients. A reduction in chemotherapy dose occurred in more patients over 80 years than those aged 70–79 years (45.1% vs. 20.1%, *p* < 0.001).

**TABLE 2:** Initial treatment regimens of patients with DLBCL (n=239).

Parameters	All patients
**Therapy**	
Chemotherapy	219 (91.6%)
Radiotherapy/Surgery	9 (3.8%)
Untreated	11 (4.6%)
**Chemotherapy dose reduction**	
Yes	53 (22.2%)
No	147 (61.5%)
Unknown	39 (16.3%)

DLBCL, diffuse large B cell lymphoma.

**TABLE 3:** Chemotherapy regimens adopted in patients with DLBCL (n=219).

Parameters	All patients n = 219	70–79 years n = 184	≥80 years n = 35
**Rituximab**			
Yes	156 (71.2%)	128 (69.6%)	28 (80.0%)
No	63 (28.8%)	56 (30.4%)	7 (20.0%)
**Regimens**			
CHOP	185 (84.5%)	162 (88.0%)	23 (65.7%)
CVP	15 (6.8%)	10 (5.4%)	5 (14.3%)
Others	19 (8.7%)	12 (6.5%)	7 (20.0%)
**Chemotherapy dose reduction**			
Yes	53 (24.2%)	37 (20.1%)	16 (45.7%)
No	147 (67.1%)	135 (73.4%)	12 (34.3%)
Unknown	19 (8.7%)	12 (6.5%)	7 (20.0%)
**Therapy cycles**			
<6	112 (51.1%)	95 (51.6%)	17 (48.6%)
≥6	107 (48.9%)	89 (48.4%)	18 (51.4%)
**Prophylaxis with G-CSF**			
Yes	44 (20.1%)	32 (17.4%)	22 (62.9%)
No	161 (73.5%)	139 (75.5%)	12 (34.2%)
Unknown	14 (6.4%)	13 (7.1%)	1 (2.9%)

DLBCL, diffuse large B cell lymphoma; CHOP, cyclophosphamide, doxorubicin, vincristine, prednisone; CVP, cyclophosphamide, vincristine, prednisone; G-CSF, granulocyte colony-stimulating factor.

### Survival Outcomes

**FIGURE 1:**
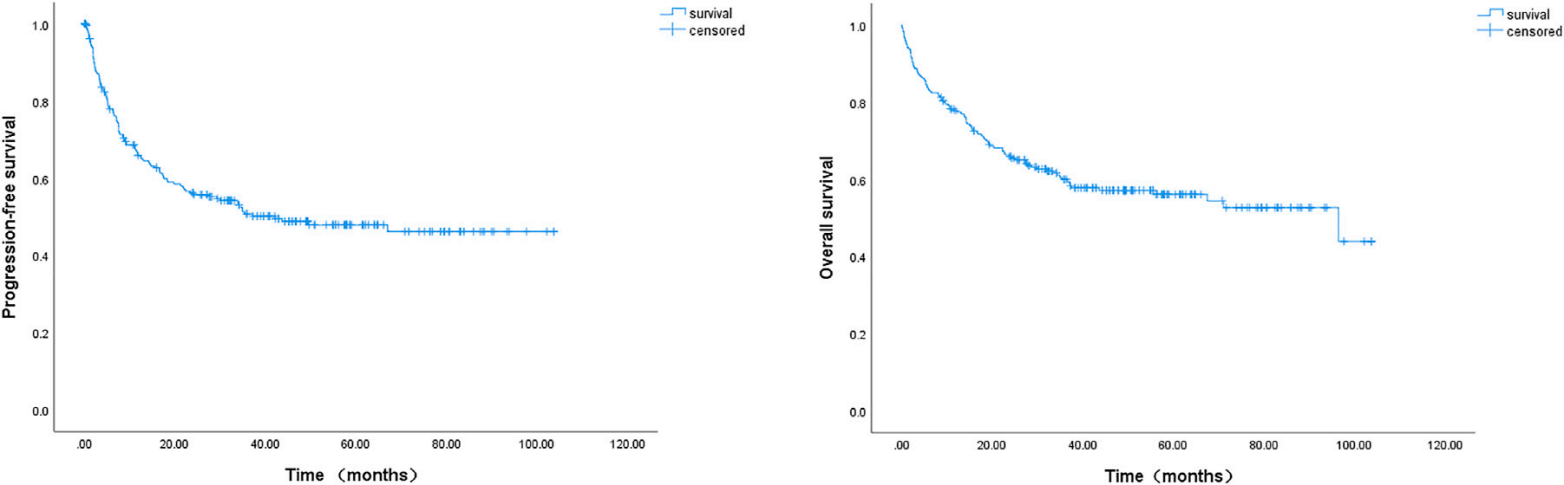
Kaplan-Meier survival curve of progression-free survival (PFS) and overall survival (OS) of 239 DLBCL patients. The 2-year PFS and 2-year OS rates were 53.0% and 65.5%, the estimated 5-year PFS and OS rates were 44.7% and 56.1%, respectively; the median PFS and median OS were 42.1 and 96.4 months, respectively.

With a median follow-up of 50 months (range, 1–102 months), the median PFS and OS were 42.1 and 96.4 months, respectively (Figure 1). The 2-year PFS and OS rates of the entire cohort were 53.0% (95% CI: 46.5–59.5%) and 65.5% (95% CI: 59.4–71.2%), respectively (Figure 1). The estimated 5-year PFS and OS rates were 44.7% (95% CI: 38.0–51.4%) and 56.1% (95% CI: 49.4–62.8%), respectively (Figure 1). Between patients younger and older than 80 years, the 2-year PFS and OS rates did not show any significant differences (data not shown): 54.5% (2-year PFS rate, 95% CI: 47.4–61.6%) and 68.0% (2-year OS rate, 95% CI: 61.5–74.5%) in the younger group; and 45.3% (2-year PFS rate, 95% CI: 29.4–61.2%) and 52.9% (2-year OS rate, 95% CI: 37.0–68.8%) in the older group, respectively.

**FIGURE 2:**
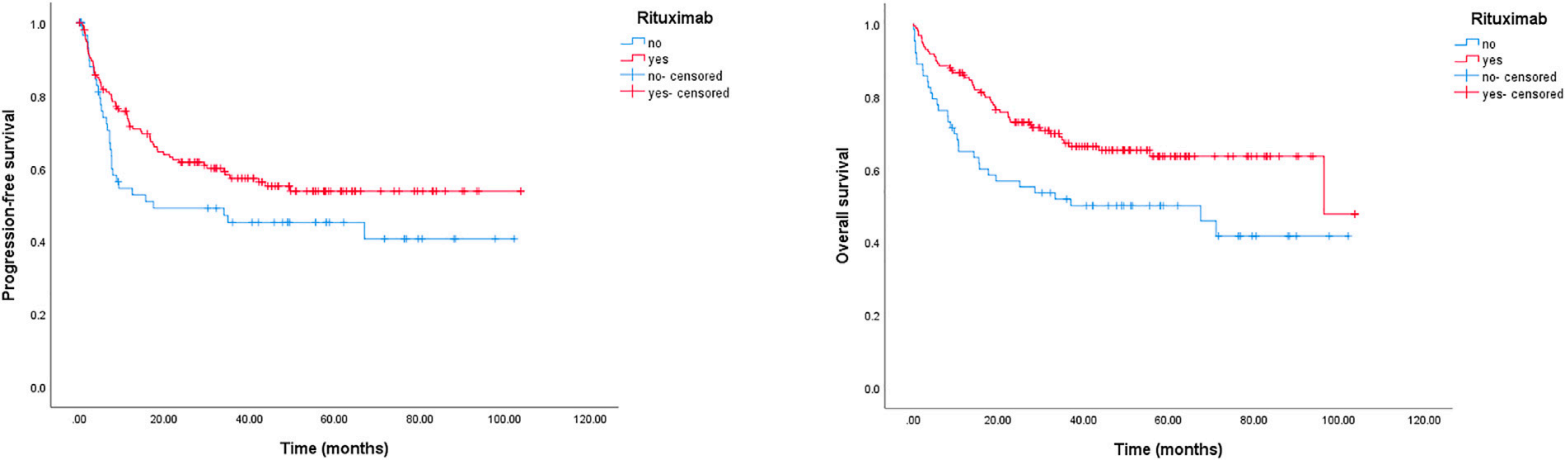
Kaplan-Meier survival curve of PFS and OS for DLBCL patients stratified by rituximab usage. The 2-year PFS rates were 61.7% (rituximab group) and 49.1% (no rituximab group), respectively, *p* = 0.082; the 2-year OS rates were 72.9% (rituximab group) and 56.8% (no rituximab group), respectively, *p* = 0.008.

In our study, the impact of rituximab on PFS and OS was also evaluated. We found that rituximab-included regimens could markedly improve OS for the entire cohort. The 2-year OS rates were 72.9% (95% CI: 65.9–80.1%) in the rituximab group and 56.8% (95% CI: 44.5–69.2%) in the non-rituximab group, and the 5-year OS rates were 63.6% (rituximab group, 95% CI: 55.2–72.0%) and 50.1% (no-rituximab group, 95% CI: 37.6–62.6%), respectively (*p* = 0.008) (Figure 2). The 2-year PFS rates were 61.7% (95% CI: 51.7–67.3%) in the rituximab-containing treatment group, and 49.1% (95% CI: 31.9–56.5%) for chemotherapy without rituximab. The estimated 5-year PFS rates were 50.5% (rituximab group, 95% CI: 42.1–58.9%) and 40.7% (no rituximab group, 95% CI: 28.4–53.0%), respectively (*p* = 0.082) (Figure 2).

**FIGURE 3:**
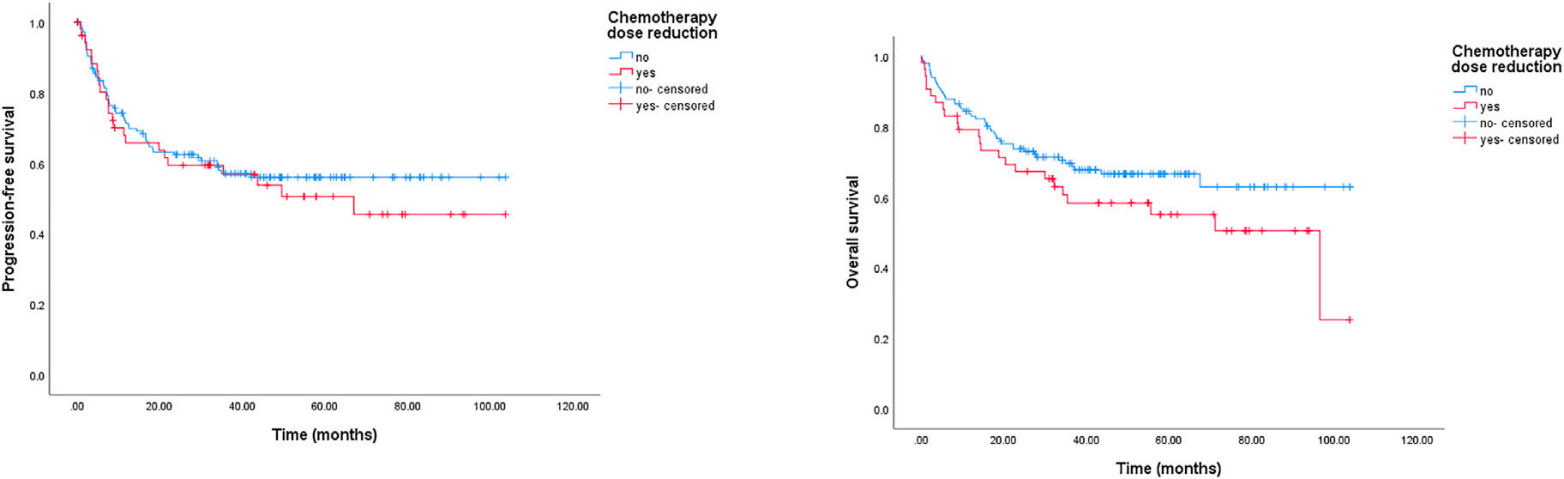
Kaplan-Meier survival curve of PFS and OS for DLBCL patients stratified by reduction in chemotherapy dose. The 2-year PFS rates were 62.4% (chemotherapy dose reduction group) and 59.4% (standard dose group), respectively, *p* = 0.567; the 2-year OS rates were 67.3% (chemotherapy dose reduction group) and 73.7% (standard dose group), respectively, *p* = 0.139.

**FIGURE 4:**
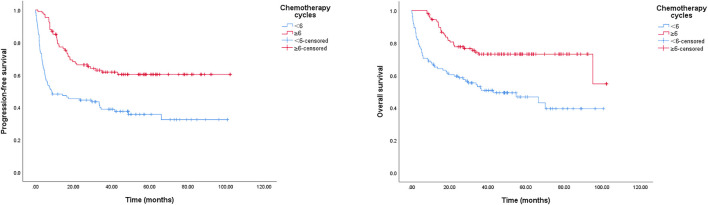
Kaplan-Meier survival curve of PFS and OS for DLBCL patients stratified by chemotherapy cycles. The 2-year PFS rates in group of < 6 chemotherapy cycles and in group of ≥ 6 cycles were 44.5%, 66.2%, *p* = 0.000; the 2-year OS rates in group of < 6 chemotherapy cycles and in group of ≥ 6 cycles were 59.5%, 77.6%, *p* = 0.000.

A reduction in chemotherapy dose was frequently delivered in clinical practice for elderly patients with DLBCL. In this study, we detected its role in survival outcomes. As shown in Figure 3, there was no significant survival difference between the standard dose group and the reduced chemotherapy dose group. However, in the group younger than 80 years, the reduced dose was associated with a significantly shorter OS, and the 2-year OS rate was 74.4% (95% CI: 67.0–81.8%) in the full dose group compared to 67.1% (95% CI: 51.8–82.4%) in the dose reduction group, *p* = 0.044 (data not shown). Meanwhile, we did not observe a marked deterioration in PFS or OS in the group aged older than 80 years with dose reduction (data not shown). Furthermore, treatment cycles was associated with survival outcomes in this study. As shown in Figure 4, patients treated with six or more cycles obtained superior OS and PFS to those treated with less than six cycles. The 2-year PFS rates were 66.2% and 44.5%, respectively (*p* = 0.000); and the 2-year OS rates were 77.6% and 59.5%, respectively (*p* = 0.000).

**FIGURE 5:**
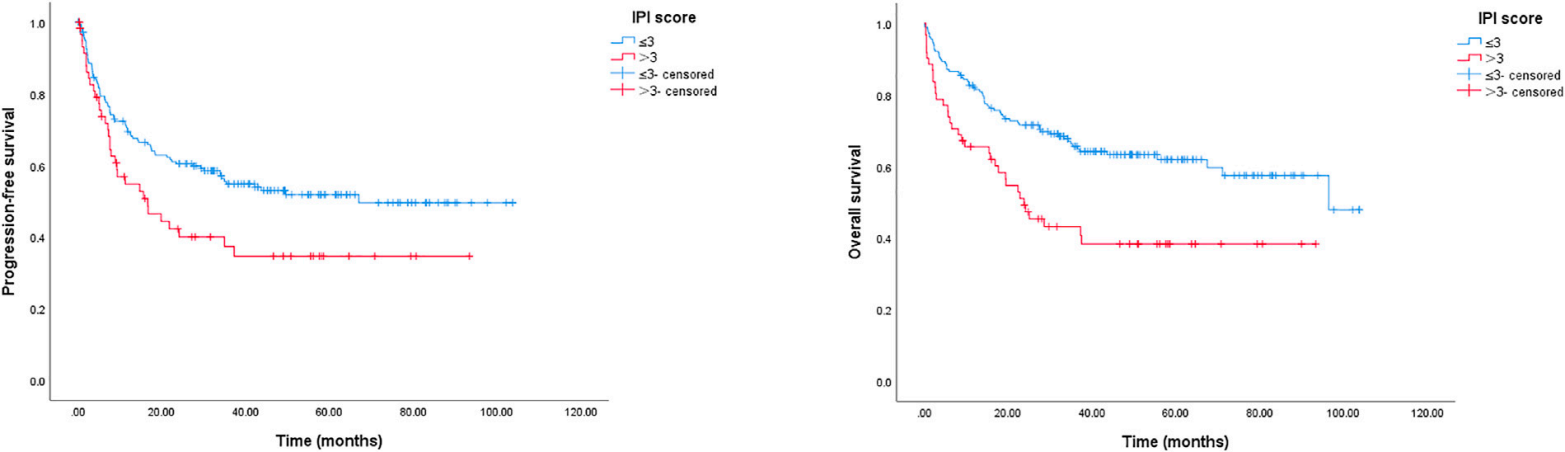
Kaplan-Meier survival curve of PFS and OS for elderly patients with DLBCL stratified by IPI score. The 2-year PFS rates were 60.5% (IPI score ≤ 3) and 42.3% (IPI score > 3), respectively, *p* = 0.021; the 2-year OS rates were 71.5% (IPI score ≤ 3) and 49.2% (IPI score > 3), respectively, *p* = 0.001.

The IPI score is frequently applied to predict the prognosis of patients with DLBCL. Here, we used the IPI score to divide patients into IPI ≤ 3 or IPI > 3 groups; as presented in Figure 5, patients with IPI ≤ 3 had longer PFS and OS than those with IPI > 3, the 2-year PFS rates were 60.5% (IPI > 3 patients) and 42.3% (IPI ≤ 3 patients), respectively (*p* = 0.021); the 2-year OS rates were 71.5% (IPI > 3 patients) and 49.2% (IPI ≤ 3 patients), respectively (*p* = 0.001).

In addition, we performed univariate and multivariate analyses in this study. From the univariate analysis results (Supplementary Table S1), an ECOG score ≥ 2 (*p* = 0.015), Ann Arbor stage III/IV (*p* = 0.012), IPI score > 3 (*p* = 0.005), and treatment without rituximab (*p* = 0.002) were significantly associated with inferior PFS. And extranodal disease involvement more than 1 site (*p* = 0.039), ECOG score ≥ 2 (*p* = 0.002), IPI score > 3 (*p* = 0.001) and treatment without rituximab (*p* = 0.000) were associated with inferior OS. Regarding the impact of GCB (germinal center B-cell like) and non-GCB subtypes on survival, we found no statistical significance for PFS and OS in this cohort. In multivariate analyses, Ann Arbor stage III/IV was significantly related to worse PFS, HR 1.640 (95% CI: 1.031–2.610, *p* = 0.037); chemotherapy dose reduction was associated with poorer OS, HR 1.825 (95% CI: 1.056–3.154, *p* = 0.031); and use of rituximab was related to OS benefit, HR 0.455 (95% CI: 0.264–0.785, *p* = 0.005) (Table 4, 5).

**TABLE 4:** Multivariate analysis of PFS in patients with DLBCL (n=239).

Parameters	HR	95% CI	p value
Stage III/IV	1.640	1.031–2.610	**0.037**
ECOG score ≥2	1.261	0.760–2.092	0.369
Serum LDH >normal	1.076	0.708–1.634	0.733
Extranodal involvement > 1 site	0.980	0.616–1.557	0.931
B symptoms	1.155	0.754–1.770	0.507
Bone marrow involvement	1.137	0.466–2.774	0.777
Rituximab	0.622	0.410–0.944	**0.026**

DLBCL, diffuse large B cell lymphoma; PFS, progression-free survival;. ECO,: eastern cooperative oncology group; GCB, germinal center B-cell like; LDH, lactate dehydrogenase; HR, hazard ratio; CI, confidence intervals.

**TABLE 5:** Multivariate analysis of OS in patients with DLBCL (n=239).

Parameters	HR	95% CI	*p* value
Stage III/IV	1.146	0.644–2.041	0.643
ECOG score ≥2	1.129	0.583–2.188	0.719
Serum LDH >normal	0.939	0.556–1.585	0.813
Extralnodal involvement > 1 site	1.266	0.716–2.237	0.418
B symptoms	1.440	0.842–2.463	0.183
Bone marrow involvement	1.275	0.363–4.473	0.704
Dose reduction	1.825	1.056–3.154	**0.031**
Rituximab	0.455	0.264–0.785	**0.005**

DLBCL, diffuse large B cell lymphoma; OS, overall survival; ECOG, eastern cooperative oncology group; GCB, germinal center B-cell like; LDH, lactate dehydrogenase; HR, hazard ratio; CI, confidence intervals.

### Toxicity

A total of 187 patients were available for the analysis of treatment-related toxicity. The most common adverse effects in this study were hematological toxicities, which accounted for 90.4%, including anemia (78.1%), leukopenia (79.7%), neutropenia (68.4%), and thrombocytopenia (31.6%) (Table 6). Additionally, nausea, vomiting, and hypoalbumin were observed in most patients. Leukopenia was the most common ≥ grade 3 event; neutropenia and febrile neutropenia occurred also in 51.9% of the patients (Table 6).

**TABLE 6:** Summary of treatment-related adverse events (n=187).

Parameters	All patients
Anemia	146 (78.1%)
Leukopenia	149 (79.7%)
Neutropenia	128 (68.4%)
Febrile neutropenia	26 (13.9%)
Thrombocytopenia	59 (31.6%)
Thrombus	9 (4.8%)
Fatigue	111 (59.4%)
Nausea/vomiting	148 (79.1%)
Hypoalbumin	128 (68.4%)
Increased aspartate aminotransferase	59 (31.0%)
Elevated creatinine	13 (5.9%)
Neuro-toxicities	6 (3.2%)
Cardio-toxicities	11 (5.9%)
Death during treatment	28 (15.0%)

## Discussion

To our knowledge, this is the largest case series to date reporting the clinical features and survival outcomes in patients with DLBCL aged ≥70 years in China. In this study, the 2-year PFS rate was 53.0% and 2-year OS rate was 65.5% for this population. The most common adverse effects were hematological toxicities, accounting for 90.4%, and leukopenia was the most frequently observed ≥ grade 3 event. Compared to a previous study reported in China, our data presented a significantly improved 5-year OS rate, which may attributed to the widespread use of rituximab, adoption of supportive therapy after chemotherapy, such as application of granulocyte colony-stimulating factor (G-CSF), and patient education, all together contributed to improve the survival of patients with DLBCL.

Rituximab has been reported to improve the PFS and OS in 60–80-years-old patients with DLBCL(Coiffier et al., 2002; Coiffier et al., 2010). In this cohort, we found similar results: rituximab increased survival outcomes compared to CHOP alone. Currently, rituximab is administered at 375 mg/m^2^ with an interval every 3 weeks for 6–8 cycles in the standard regimen. Furthermore, some studies have investigated whether an increase in rituximab dose could improve treatment efficacy in elderly patients, showing that 500 mg/m^2^ of rituximab improved PFS and OS for elderly male cases compared to standard dose (Pfreundschuh et al., 2014; Pfreundschuh et al., 2017; He et al., 2021). No patients received this maximum dose of rituximab in this study. In the future, a potential research direction involves exploring the application of rituximab intensification regimens in specific population. Furthermore, in clinical practice, eight cycles of rituximab plus six cycles of CHOP21 or six cycles of R-miniCHOP are recommended to treat DLBCL in elderly patients according to some critical study results (Coiffier et al., 2010; Pfreundschuh et al., 2008; Delarue et al., 2013; Sehn et al., 20182018; Wц╓sterlid et al., 2018). From our data, in contrast to ≥ 6-cycle treatment regimens, patients receving less than 6 cycles presented lower PFS and OS, suggesting that 6 or 8 cycles of treatment are necessary in clinical practice for this population.

The treatment strategy for elderly patients with DLBCL has not been well established. In comparison with R-CHOP14 applied to DLBCL patients aged 60–80 years, R-CHOP21 could achieve similar efficacy and decreased the frequency and severity of hematological adverse effects and the number of red blood cell transfusions, making R-CHOP21 a frequently administered regimen (Cunningham et al., 2013; Delarue et al., 2013). In our study, 84.5% of patients treated with R-CHOP21, 74.4% of whom achieved full dose in patients aged 70–79 years and 67.1% in those older than 80 years. R-miniCHOP is recommended in cases ≥ 80 years (Peyrade et al., 2011; Eyre et al., 2019; NCCN, 2022). Peyrade et al. reported that European patients aged 80 years treated with R-miniCHOP obtained a 2-year OS rate of 59% and a 2-year PFS rate of 47% (Peyrade et al., 2011). From our data, we observed similar results in the Chinese population with a 2-year OS rate of 52.9% and a 2-year PFS rate of 45.3% in patients treated with full-dose or reduced-dose therapy. Furthermore, in our subgroup analysis, we found that a reduced chemotherapy dose was not correlated with an improved 2-year OS rate when compared to the standard dose in the population older than 80 years (data not shown), which was comparable to the results of previously studies (Peyrade et al., 2011; Eyre et al., 2019). However, in patients aged 70–79 years, chemotherapy dose reduction (≥20%) could have a worse impact on OS than full-dose regimens. According to a previous study (Meguro et al., 2012), which reported that 70% of the standard dose would influence OS and PFS in patients over 70 years of age. Taken together, based on our data, the standard dose should be administered in 70–79-year-old patients with DLBCL in China if possible, and R-miniCHOP is reasonable for patients over 80 years of age.

DLBCL is divided into GCB, ABC and unclassified subtypes based on a gene expression profiling (GEP) (Scott et al., 2014). Furthermore, the Hans algorithm had a prognostic value based on the determination of the cell of origin (COO) (Hans et al., 2004). In our study, we applied the Hans algorithm to divide patients into GCB and non-GCB subtypes, with 21.8% of the GCB subtype and 61.9% of the non-GCB subtype. Lenz et al. reported that the GCB subtype had superior PFS and OS rates to the ABC subtype with R-CHOP(Lenz et al., 2008). Our cohort found that the GCB subtype had a similar survival outcome compared to the non-GCB subtype (data not shown). Moreover, in the univariate analyses, patients with GCB or non-GCB had similar OS and PFS.

The IPI score is a widely used prognostic model in DLBCL. Recently, a study reported that the National Comprehensive Cancer Network (NCCN)-IPI had the greatest absolute difference in OS estimates between the highest- and lowest-risk groups and the best-discriminated OS compared to the IPI score and the age-adjusted IPI (aaIPI) for the entire cohort of DLBCL(Ruppert et al., 2020). In our study, NCCN-IPI and aaIPI were not suitable to evaluate prognosis, as the included patients were all aged ≥ 70 years. According to the IPI score, there are significant differences in PFS and OS between the IPI ≤ 3 and IPI > 3 groups, suggesting that the IPI score could predict the prognosis in elderly patients. Recently, a study from Italy reported that combining the comprehensive geriatric assessment (CGA) with the IPI score could identify three risk groups with notable differences in terms of OS (Spina et al., 2019; Merli et al., 2021), which would help clinicians to evaluate the prognosis of patients with greater precisely.

This study had several limitations. First, our study was retrospective, and selection and information bias could not have been avoided. Second, patients in our study did not undergo the CGA, which could have contributed to better predict OS and assist clinicians in determining whether or not to treat patients with full-dose chemotherapy (Hamaker et al., 2014; Lin et al., 2017).

## Conclusion

Our study presents the clinical profiles and survival outcomes of elderly patients with DLBCL in China. Treatment of these patients requires careful evaluation of toxicities and benefits. To this end, a prognosis model, such as comprehensive geriatric assessment, is required in clinical practice to optimally manage elderly patients with DLBCL.

## Data Availability

The raw data supporting the conclusions of this article will be made available by the authors, without undue reservation.
