# Supplementary material for: A Multicenter Study of 239 Patients Aged Over 70 Years With Diffuse Large B-Cell Lymphoma in China
Source: Front Pharmacol. 2022 Jul 18;13:953808. doi: 10.3389/fphar.2022.953808 (PMC9341248; doi:10.3389/fphar.2022.953808)
Supplement: Supplementary file 1 [file Table1.docx]

**Supplementary table 1. Univariate analysis of PFS and OS in patients with DLBCL (n=239).**

| **Parameters** | **All patients**  **n=239** | **2-year PFS (%)** | **95% CI** | ***p* value** | **2-year OS**  **(%)** | **95% CI** | ***p* value** |
| --- | --- | --- | --- | --- | --- | --- | --- |
| **Age** |  |  |  | 0.542 |  |  | 0.238 |
| 70-79 years | 200 | 54.5 | 47.4-61.6 |  | 68.0 | 61.5-74.5 |  |
| ≥80 years | 39 | 45.3 | 29.4-61.2 |  | 52.9 | 37.0-68.8 |  |
| **Sex** |  |  |  | 0.512 |  |  | 0.908 |
| Male | 143 | 52.6 | 44.5-60.8 |  | 66.6 | 58.8-74.4 |  |
| Female | 96 | 53.5 | 43.3-63.7 |  | 63.9 | 54.1-73.7 |  |
| **ECOG score** |  |  |  | **0.015** |  |  | **0.002** |
| 0-1 | 194 | 56.0 | 48.9-63.1 |  | 69.4 | 62.7-76.1 |  |
| ≥2 | 45 | 40.0 | 25.7-54.3 |  | 48.9 | 34.2-63.6 |  |
| **B symptoms** |  |  |  | 0.127 |  |  | 0.128 |
| Yes | 65 | 44.2 | 32.1-56.4 |  | 56.6 | 44.5-68.8 |  |
| No | 174 | 56.3 | 48.9-63.8 |  | 68.9 | 61.8-76.0 |  |
| **Hans subtype** |  |  |  | 0.211 |  |  | **0.046** |
| GCB | 52 | 56.8 | 43.1-70.5 |  | 72.2 | 59.9-84.6 |  |
| non-GCB | 148 | 53.8 | 45.8-61.8 |  | 67.2 | 59.6-74.8 |  |
| Unknown | 39 | 45.7 | 30.0-61.4 |  | 50.1 | 34.0-66.2 |  |
| **Ki-67 index** |  |  |  | 0.05 |  |  | 0.142 |
| ≤90% | 192 | 56.1 | 49.0-63.2 |  | 68.0 | 61.3-74.7 |  |
| ＞90% | 7 | 28.6 | -4.9-62.1 |  | 57.1 | 20.5-93.8 |  |
| Unknown | 40 | 42.5 | 27.2-57.8 |  | 55.0 | 39.5-70.5 |  |
| **Number of extra-nodal involvement** |  |  |  | 0.161 |  |  | **0.039** |
| 0 or 1 | 174 | 57.2 | 49.8-64.7 |  | 70.9 | 64.0-77.8 |  |
| ≥2 | 65 | 41.3 | 29.0-53.7 |  | 50.6 | 38.1-63.1 |  |
| **Bone marrow involvement** |  |  |  | 0.068 |  |  | 0.065 |
| Yes | 12 | 31.3 | 3.9-58.7 |  | 40.0 | 11.4-68.6 |  |
| No | 227 | 54.0 | 47.5-60.5 |  | 66.8 | 60.5-73.1 |  |
| **Ann Arbor stage** |  |  |  | **0.012** |  |  | 0.108 |
| I-II | 96 | 63.3 | 53.7-72.9 |  | 70.4 | 61.2-79.6 |  |
| III-IV | 143 | 46.0 | 37.8-54.2 |  | 62.3 | 54.3-70.3 |  |
| **Bulky disease** |  |  |  | 0.860 |  |  | 0.411 |
| Yes | 38 | 57.7 | 41.8-73.6 |  | 60.0 | 44.3-75.7 |  |
| No | 201 | 52.1 | 45.0-59.2 |  | 66.5 | 59.8-73.2 |  |
| **Serum LDH** |  |  |  | 0.194 |  |  | 0.27 |
| Normal | 102 | 58.5 | 48.9-68.1 |  | 70.2 | 61.2-79.2 |  |
| Elevated | 137 | 48.8 | 40.4-57.2 |  | 62.0 | 53.8-70.2 |  |
| **IPI score** |  |  |  | **0.005** |  |  | **0.001** |
| 1-3 | 178 | 58.7 | 51.5-66.0 |  | 71.5 | 64.8-78.2 |  |
| 4-5 | 61 | 35.7 | 23.4-48.1 |  | 47.3 | 34.4-60.2 |  |
| **Comorbidities** |  |  |  | 0.477 |  |  | 0.472 |
| Yes | 205 | 54.2 | 47.3-61.1 |  | 66.8 | 60.3-73.3 |  |
| No | 34 | 45.3 | 28.1-62.6 |  | 57.2 | 40.2-74.3 |  |
| **Rituximab** |  |  |  | **0.002** |  |  | **0.000** |
| Yes | 156 | 59.5 | 51.7-67.4 |  | 72.9 | 65.8-80.0 |  |
| No | 83 | 40.7 | 30.1-51.3 |  | 51.5 | 40.7-62.3 |  |
| **Chemotherapy dose reduction** |  |  |  | **0.000** |  |  | **0.000** |
| Yes | 53 | 56.0 | 42.5-69.5 |  | 67.3 | 54.6-80.0 |  |
| No | 147 | 60.1 | 52.1-68.1 |  | 73.7 | 66.5-81.0 |  |
| Unknown | 39 | 21.9 | 8.6-35.2 |  | 32.3 | 17.4-47.2 |  |
| **Regimens** |  |  |  | **0.000** |  |  | **0.000** |
| CHOP | 185 | 58.5 | 51.4-65.6 |  | 72.6 | 66.1-79.1 |  |
| CVP | 15 | 66.0 | 41.7-90.3 |  | 64.3 | 39.2-89.4 |  |
| others | 39 | 21.9 | 8.6-35.2 |  | 32.3 | 17.4-47.2 |  |

OS: overall survival. PFS: progression-free survival. DLBCL: diffuse large B cell lymphoma. ECOG: eastern cooperative oncology group. GCB: germinal center B-cell like. LDH: lactate dehydrogenase. IPI: international prognostic index. CHOP: cyclophosphamide, doxorubicin, vincristine, prednisone. CVP: cyclophosphamide, vincristine, prednisone.
